# Alignment Effects in Spatial Perspective Taking from an External Vantage Point

**DOI:** 10.3390/brainsci11020204

**Published:** 2021-02-07

**Authors:** Adamantini Hatzipanayioti, Marios N. Avraamides

**Affiliations:** 1 Centre for Tactile Internet with Human-in-the-Loop (CeTI), Technische Universität, 01062 Dresden, Germany; 2 Chair of Lifespan Developmental Neuroscience, Faculty of Psychology, Technische Universität, 01062 Dresden, Germany; 3 CYENS—Centre of Excellence, 1066 Nicosia, Cyprus; mariosav@ucy.ac.cy; 4 Department of Psychology & Center for Applied Neuroscience, University of Cyprus, 1678 Nicosia, Cyprus

**Keywords:** spatial memory, virtual reality, alignment effect, perceptive taking

## Abstract

In three experiments, we examined, using a perceptual task, the difficulties of spatial perspective taking. Participants imagined adopting perspectives around a table and pointed from them towards the positions of a target. Depending on the condition, the scene was presented on a virtual screen in Virtual Reality or projected on an actual screen in the real world (Experiment 1), or viewed as immediate in Virtual Reality (Experiment 2). Furthermore, participants pointed with their arm (Experiments 1 and 2) vs. a joystick (Experiment 3). Results showed a greater alignment effect (i.e., a larger difference in performance between trials with imagined perspectives that were aligned vs. misaligned with the orientation of the participant) when executing the task in a virtual rather than in the real environment, suggesting that visual access to body information and room geometry, which is typically lacking in Virtual Reality, influences perspective taking performance. The alignment effect was equal across the Virtual Reality conditions of Experiment 1 and Experiment 2, suggesting that being an internal (compared to an external) observer to the scene induces no additional difficulties for perspective taking. Equal alignment effects were also found when pointing with the arm vs. a joystick, indicating that a body-dependent response mode such as pointing with the arm creates no further difficulties for reasoning from imagined perspectives.

## 1. Introduction

Many tasks of everyday life require that we mentally adopt a spatial perspective other than the one we occupy physically. For example, in order to describe a turn as left or right when providing route directions to others, we may imagine ourselves at a location on the route, facing a particular direction. This mental perspective taking is rather effortful, as documented by the presence of an *alignment effect*: localizing an object from an imagined perspective is slower and more prone to error as the angular disparity (i.e., the difference between one’s actual and imagined perspective) increases [1,2,3,4,5,6,7,8,9]. The goal of the present study is to further investigate the difficulties observed in perspective taking, as indexed by the size of the alignment effect, using a perceptual task in various testing conditions.

One account for the alignment effect is that it reflects processing costs associated with the mental transformation required to adopt an imagined perspective [4]. That is, in order to localize an object from an imagined perspective, one needs to first mentally rotate an egocentric reference frame in alignment with the imagined perspective and then use it to compute the location of the object from that orientation. According to this *mental transformation account*, the greater the angle of rotation, the longer it takes to complete this process and the higher the probability of committing an error.

An alternative *sensorimotor interference account* introduced by May [10] posits that the main source of the alignment effect is the presence of spatial conflicts at the time of response computation and execution. According to May [10], when responding from an imagined perspective, difficulties arise because of the need to inhibit egocentric (i.e., self-to-object) codes that specify where objects are relative to the observer’s actual position and orientation. These self-to-object codes are established when experiencing a spatial layout and are automatically activated with the presentation of a target object. The activated codes specify where an object is relative to the actual position and orientation of the observer, causing interference to the computational processes that are carried out in order to compute and execute a response from an imagined perspective (see also Avraamides and Kelly [11]).

The mental transformation and the sensorimotor interference accounts place the locus of the alignment effect at different points in the information processing stream. On one hand, the mental transformation account places the difficulty of reasoning from imagined perspectives at the early stage of adopting the perspective. On the other hand, the sensorimotor interference account posits that the difficulty stems from the later stage of response computation and execution.

Notably, the two accounts are not mutually exclusive. In fact, the results from a study by Sohn and Carlson [6] suggest that both mental transformation and response computation/execution processes contribute to the alignment effect by showing that difficulties in responding from imagined perspectives arise both during the process of adopting the imagined perspective and during the response computation/execution stage. Using a perceptual task, Sohn and Carlson [6] presented participants with a schematic round table on a computer screen. In each trial, participants had to adopt a perspective around the table, indicated by a green circle, and localize from it a target, marked as a red circle. Depending on the condition, either the perspective or the target information was presented first with varying extents of Stimulus-Onset-Asynchrony (SOAs of 0, 200, 400, 600 and 800 ms). Results from a first experiment in which responses were made by pressing keys mapped to egocentric verbal labels (e.g., “near left”), showed that when perspective information was provided ahead of the target, the alignment effect was reduced with increasing SOAs. That is, when participants had sufficient time to adopt the imagined perspective before the target was presented, they became faster to respond from misaligned perspectives. This finding provides support to the claim of the mental transformation account that the alignment effect arises during the process of adopting the imagined perspective. However, in a follow-up experiment in which response keys were mapped to non-spatial responses (i.e., the letters W, S, L and P were arbitrarily assigned to a target location), the alignment effect was completely eliminated with advance perspective information. This result supports the sensorimotor interference account by suggesting that interference from spatial stimulus–response mappings may also contribute to the alignment effect.

Whereas a number of past studies have attempted, with varied success, to reduce the alignment effect by providing advance perspective information to aid mental transformation (e.g., May [10]; Wang [12]), beyond Sohn and Carlson [6], not much research has attempted to reduce sensorimotor interference (but see Avraamides et al. [13]). In the current study, we attempt to do so by blocking visual access to one’s body and response medium. The idea is that if sensorimotor interference is present because of the spatial correspondence between spatial locations and responses [14], making that correspondence less salient by blocking vision to one’s body and arms could reduce interference and improve performance from imagined perspectives.

Although not having visual access to our own body is highly uncommon in everyday life, it is the typical scenario for Virtual Reality (VR) experiences. With consumer VR gear, the user navigates and/or interacts with a simulated environment without seeing his/her own body and arms. In most VR systems, users only see a representation of their hands. Past studies show that virtual hands may increase the sense of ownership in VR [15], although users do not readily recognize the virtual hands as their own [16]. Therefore, an interesting question arises: can responding from an imagined perspective be made more efficient in VR, where visual information about one’s body is lacking?

The present study addresses this question by comparing perspective taking performance across conditions in which a spatial layout, modelled after the one used by Sohn and Carlson [6], is experienced either in Virtual Reality, where participants cannot see their own body and arms, or in the real world, where they can. The task required that participants imagine themselves occupying various positions around a round table and indicate by pointing with their arm, to the position of a virtual character also sitting around the table. As in Sohn and Carlson [6], in a real world condition, the task was displayed on a 2D screen; however, instead of a computer screen, we used a large projection screen. In the VR equivalent, the task was carried out on a project screen in the virtual environment. If blocking visual access to one’s body and the actual environment reduces sensorimotor interference, a smaller alignment effect is expected when the task is carried out in the virtual than in the real world.

## 2. Experiment 1

Experiment 1 involved two testing conditions. In the *Virtual Environment (VE)* condition, participants were immersed in a virtual room that contained a projector screen. The spatial scene was presented on this virtual screen. In contrast, in the *Real Environment (RE)* condition, the spatial scene was presented on an actual projector screen in the laboratory. If the absence of visual information about one’s body and the surrounding environment in VR makes reasoning from imagined perspectives easier, then we expect a reduced alignment effect in the VE compared to the RE condition.

### 2.1. Method

#### 2.1.1. Participants

Fifty students from the University of Cyprus participated in the experiment in exchange for course credit. Twenty-six participants were randomly assigned to the RE condition and twenty-four participants to the VE condition.

#### 2.1.2. Materials, Stimuli and Apparatus

**Figure 1:**
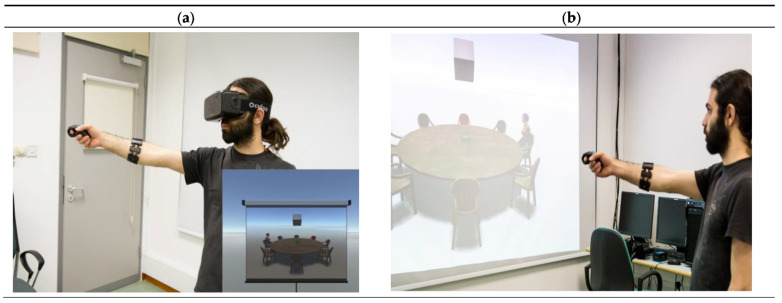
Example of a participant carrying out the pointing task in the Virtual Environment (VE) condition (**a**) and the Real Environment (RE) condition (**b**) of Experiment 1. In the VE condition, the participant is immersed in a virtual environment that contains a projector screen. The spatial scene is represented as 2D images on the virtual screen. In the RE condition, the spatial scene is presented as a 2D image on an actual projector screen in the laboratory.

Stimuli constituted of a virtual environment depicting a round table with 7 empty sitting positions, one highlighted empty seat and a female virtual character sitting on one of the 8 seats. The highlighted seat indicated the imagined perspective to be adopted, whereas the virtual character served as the target towards which participants pointed from the imagined perspective. We used a human character as the target in order to make the task more similar to the everyday life scenario of sitting around a table with others. Participants in the VE condition viewed the scene as a 2-dimensional projection on a virtual screen (Figure 1a). For this condition, an Oculus Rift DK2 head-mounted display was used with a 960 × 1080 resolution per eye and a 75 Hz refresh rate. Participants in the RE condition viewed the layout on a projector screen within the laboratory (Figure 1b) and carried out pointing while standing at the center of the laboratory room. The distance between the observer and the screen in the real and virtual environments was 2.5 m in both experimental conditions. Additionally, in both conditions, participants had a fixed body and head orientation that enabled them to have a forward view of the scene. A script written in the Unity game engine was used to present the virtual content and control the experiment during the testing phase. Participants pointed with their dominant arm and clicked a handheld air mouse to log their response. The orientation of their arm was tracked with 1⁰ accuracy by a Myo Armband (Thalmic Labs, Kitchener, ON, Canada) that participants wore on their arm. A recent study that used this device in rehabilitation game scenarios has found that the electromyographic sensors, the accelerometer and the gyroscope that the armband includes trace gestures and motion with high accuracy and precision [17].

In addition to the experimental task, participants completed the revised version of the Spatial Orientation Test (SOT; [18,19]) and filled out the Santa Barbara Sense of Direction Scale (SBSOD) questionnaire [20]. The Spatial Orientation Test is a paper and pencil tool that measures perspective taking. It requires participants to draw a line indicating how they would point to an object from an imagined perspective in a schematic scene with drawings of objects. The Santa Barbara Sense of Direction Scale (SBSOD) is a self-report measure of environmental spatial ability. We correlated performance on the main task with the scores from these tests in order to verify that the participants carried out the task using spatial transformations rather than non-spatial strategies (e.g., counting positions around the table). Given the small sample size of each experiment, we have run these correlations by collapsing the data across the 3 experiments.

#### 2.1.3. Design

The experiment adopted a 2 (environment condition: VE vs. RE) × 7 (imagined perspective: physical perspective 0° vs. misaligned perspectives) mixed factorial design with imagined perspective manipulated within participants and environment condition between.

#### 2.1.4. Procedure

Participants signed an informed consent form before the start of the experiment and completed the Santa Barbara Sense of Direction Scale (SBSOD) and the Spatial Orientation Test. (Correlations between pointing error and latency in the perspective taking task and scores on the SOT and the SBSOD are presented together for the three experiments in the Results section of Experiment 3.)

Prior to the experimental phase, participants in the VE condition were familiarized with the virtual environment and were provided with instructions about pointing. Participants wore the head-mounted-display and, at first, they were asked to point with their arm towards the position of a chair that appeared at various locations in the virtual environment using the Myo Armband and the air mouse. Then, each participant carried out a practice block of 10 trials, where each trial was randomly chosen by an algorithm from the set of all possible combinations of imagined perspectives and target locations (i.e., 7 imagined perspectives × 8 targets). Participants in the RE condition followed the same procedure but the experimental trials were presented on a projection screen within the laboratory.

Upon completing the practice trials, participants proceeded to the experimental trials. In each trial, a round table was presented with a female virtual character sitting on one of the 8 seats. The other 7 seats remained empty but one of them turned red. Participants were asked to imagine sitting on the highlighted chair facing the center of the table and point from that perspective with their arm towards the female virtual character. They were instructed to point as fast as possible without sacrificing accuracy for speed. Pointing response and response latency were recorded by the computer and analyzed offline. Response latency was measured from the onset of the trial until participants pressed the trigger of the handheld controller to enter their pointing response. All participants carried out 2 blocks of 56 trials each. Each block consisted of every single combination of imagined perspectives and targets including trials that were aligned and misaligned. The task was identical in the two conditions and the only difference was that participants in the VE condition stood in a virtual room that contained a projector screen onto which the task was presented while those in the RE stood in the center of an actual room and were presented with the task on a real projector screen.

### 2.2. Results

Data were analyzed using Mixed Model Analyses of Variance (ANOVAs) that were carried out separately for pointing error and response latency. In order to assess the presence of alignment effects (i.e., the difference in performance on trials that were aligned with the physical perspective compared to those who were not) we followed the approach of Kelly et al. (2007) to average the misaligned perspectives to a single mean and compare it with the physical (0°) perspective. Therefore, imagined perspective (physical perspective 0° vs. misaligned perspectives) was analyzed as the within-participants factor and environment condition (VE vs. RE) as the between-participants factor. Pointing errors greater than 75° and response times deviating more than 3 standard deviations from the mean of each participant were considered outliers and were removed from the analyses.

#### 2.2.1. Pointing Error

**Figure 2:**
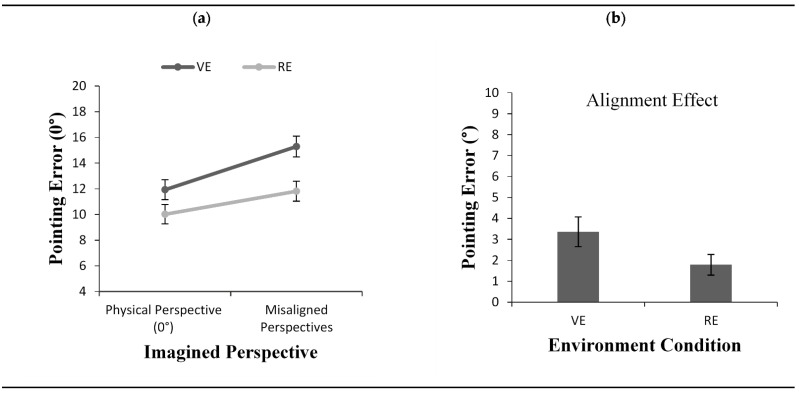
Pointing error (**a**) as a function of imagined perspective and alignment effect for pointing error (**b**) across environment conditions (VE vs. RE) in Experiment 1. Error bars represent standard errors from the ANOVA and from a *t*-test, respectively.

Results revealed a main effect of imagined perspective, *F*(1, 48) = 36.45, *p* < 0.001, *η*^2^ = 0.43. As shown in Figure 2a, the error was smaller when the imagined perspective at testing was aligned with participants’ physical perspective compared to the remaining misaligned imagined perspectives. Results also revealed a significant main effect for the environment condition in that the pointing error was greater in the VE condition (M = 13.61) than the RE condition (M = 10.91), *F*(1, 48) = 7.09, *p* < 0.05, *η*^2^ = 0.12. However, although the alignment effect was numerically smaller in the RE condition (Figure 2b), the interaction between the environment condition and the imagined perspective did not reach significance, *F*(1, 48) = 3.41, *p* = 0.07, *η*^2^ = 0.06.

#### 2.2.2. Response Latency

**Figure 3:**
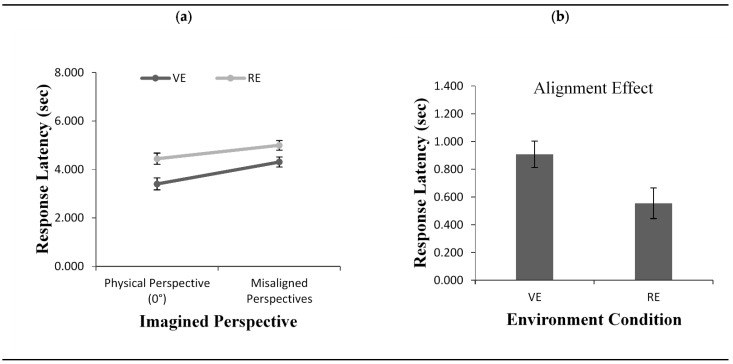
Response Latency (**a**) as a function of imagined perspective and alignment effect for response latency (**b**) across environment conditions (VE vs. RE) in Experiment 1. Error bars represent standard errors from the ANOVA and from a *t*-test, respectively.

Participants’ response latency also depended on the alignment of the imagined perspective, *F*(1, 48) = 99.64, *p* < 0.001, *η*^2^ = 0.67 (Figure 3a). Overall, participants responded faster when the imagined perspective adopted at testing was aligned with their physical perspective compared to the remaining imagined perspectives. The results also revealed a significant main effect for environment condition, *F*(1, 48) = 7.98, *p*< 0.01, *η*^2^ = 0.10. Participants were faster to respond in the VE condition (M = 3.85) than in the RE condition (M = 4.72). Most importantly, the results showed a significant interaction between imagined perspectives and environment condition *F*(1, 48) = 5.80, *p* < 0.05, *η*^2^ = 0.10. The interaction was caused by the presence of a smaller alignment effect in the RE (M = 0.55) than in the VE (M = 0.90) condition, *t*(48) = 2.40, *p* = 0.02 (Figure 3b).

### 2.3. Discussion

The results from Experiment 1 showed that overall participants were faster, but at the same time less accurate, in the VE condition compared to the RE condition. We believe this speed–accuracy trade-off was caused by the fact that, in the real-world testing, participants could see their arm, which allowed them to fine tune their manual responses at the expense of reaction time.

More importantly, and against our expectations, the alignment effect was greater in the VE condition compared to the RE condition. We expected that blocking access to one’s body in the virtual world would reduce the alignment effect by making self-to-object codes less salient. However, it could be that the VE has, instead, made self-to-object codes more salient by providing more immersion than in the real world. Indeed, the design of VR environments has some qualities that increase immersion (e.g., wild field of view, continuity of the surrounding, exploration of the scene in all directions; [21]) and provides the necessary information (i.e., spatial reference frames) that encourages egocentric encoding in visual-spatial tasks [22,23]. Therefore, participants in the VE condition might have felt more connected to the virtual scene and might have found it harder to ignore or suppress any conflicting self-to-object spatial codes when pointing from an imagined perspective. In the case of the RE condition, which, by default, lacks some of the qualities of immersive VR, participants could see their body, and perhaps this helped to decontextualize them from the spatial scene, making more salient the fact that they were external to the projected layout and not immersed to it. In contrast, experiencing the projected layout in VE, without visual access to their body and objects in the laboratory, might have blurred the boundaries between their body and the spatial scene.

Overall, it seems that VE hinders perspective taking tasks, at least when the task is perceptual. If viewing a projected layout in Virtual Reality creates greater conflicts than viewing it in the real world, then a question that arises is whether situations in which one is internal to the spatial layout would pose even greater difficulties for perspective taking, yielding a greater alignment effect compared to situations where the observer is external to the scene. Studies using memory tasks have shown that alignment effects are more pronounced when reasoning about environments that are immediate to the observer compared to remote ones, possibly because, in the latter case, the observer is decontextualized from the layout and no sensorimotor interference is present [13,24]. In one study, for example, Avraamides et al. [13] had participants memorizing a layout of objects that surrounded them in a virtual environment and then they pointed to objects’ locations either within the same virtual environment or after being transported to a different virtual environment. The results showed that when participants were tested in a different environment, the alignment effect was reduced and was even eliminated it for some angles compared to testing in the same environment. Based on these findings, Avraamides et al. [13] argued that, perhaps, when we are being surrounded by objects, sensorimotor influences emerging from knowing the real location of objects relative to our position in space hinder performance when reasoning about imagined perspectives. In contrast, as one’s body is detached from the layout—such as when reasoning about remote environments—sensorimotor conflicts have less or no influence in adopting imagined perspectives (see Avraamides and Kelly [11] for an extensive discussion). In the conditions we tested in Experiment 1, the layout was projected on a screen, either real or virtual, and in both cases the participant was an external observer to the layout. Thus, in both conditions we tested, the layout could be considered as remote. However, in contrast to past studies that relied on memory tasks, in our experiment participants had continuous visual access to the locations of objects, which could have contributed to sensorimotor interference.

In Experiment 2, we test whether spatial difficulties resulting in strong alignment effects are more pronounced when the layout is perceived as immediate rather than as remote. It could be that egocentric codes, which are automatically activated with the presentation of the stimulus and define the spatial relations between the observer and the objects, are even greater when one perceives the objects of the layout as immediate (e.g., when being adjacent to the layout) in immersive VR than when the same scene is viewed through a medium (e.g., as with the virtual projector as in Experiment 1). Thus, in Experiment 2, we run a Virtual Reality condition in which participants carry out the same task as in Experiment 1 but with the stimuli presented as part of the virtual environment they are in.

## 3. Experiment 2

The goal of Experiment 2 was to investigate whether a greater alignment effect—indicative of more pronounced sensorimotor conflicts—would be present when reasoning about an immediate perceptual scene. Similar to Experiment 1, participants in Experiment 2 had to imagine themselves occupying various positions around a round table and indicate by pointing the position of a virtual character also sitting around the table. To equate conditions with the VE condition of Experiment 1 as much as possible, the layout was experienced using virtual reality. Unlike Experiment 1, where the layout was presented to participants on a virtual projector within VR, in Experiment 2 the layout was presented in 3D and adjacent to participants. That is, while standing in VR, participants could view the layout at a distance of 2.5 m directly in front of them. To differentiate the two conditions, we will refer to the VE condition of Experiment 1 as the *Remote VE* and the condition of Experiment 2 as the *Immediate VE* condition. If the alignment effect in the Remote VE condition of Experiment 1 was primarily caused by sensorimotor interference due to high degree of immersion compared to the real word, then perhaps the alignment effect would be even greater with the immediate scene presented in immersive VR.

### 3.1. Method

#### 3.1.1. Participants

Twenty-four students from the University of Cyprus participated in the experiment in exchange for course credit. None of the participants had previously carried out Experiment 1. As in Experiment 1, pointing errors that were off by more than 75° from the correct position, and response latencies deviating more than 3 standard deviations from the mean of each participant, were removed from the analyses.

#### 3.1.2. Materials and Procedure

**Figure 4:**
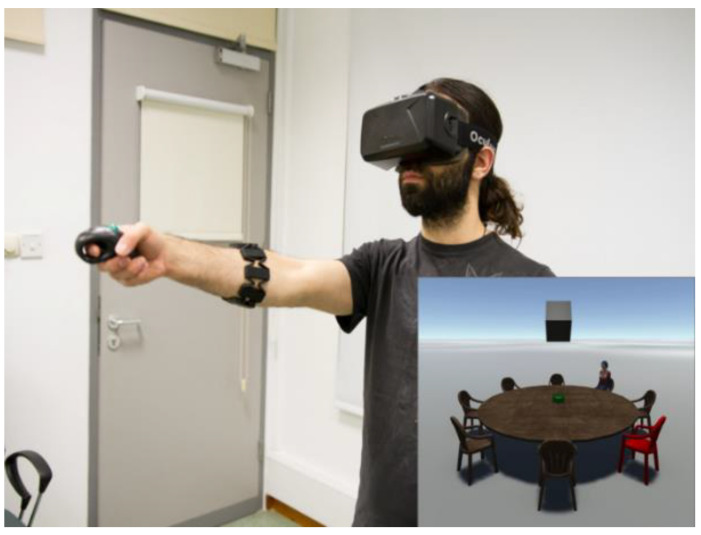
Example of a participant carrying out the task in the Immediate VE condition of Experiment 2. The spatial scene appears in 3D in front of the participant in the same virtual environment.

The materials and procedure were identical to those of Experiment 1, with one notable exception. While in the Remote VE condition of Experiment 1 participants were external observers to a layout projected on a screen, in Experiment 2, participants were adjacent to that layout (Figure 4).

### 3.2. Results

To investigate the effect of doing the task in VR vs. the real world, we analyzed the data from Experiment 2 together with the data from the Remote VE condition of Experiment 1. Due to multiple testing using the same data set of Experiment 1, we used a Bonferroni correction for the analysis. We carried out separate Mixed Model ANOVAs for pointing error and response latency, with imagined perspective as the within-participants factor and environment condition (Immediate VE vs. Remote VE) as the between-participants factor.

#### 3.2.1. Pointing Error

**Figure 5:**
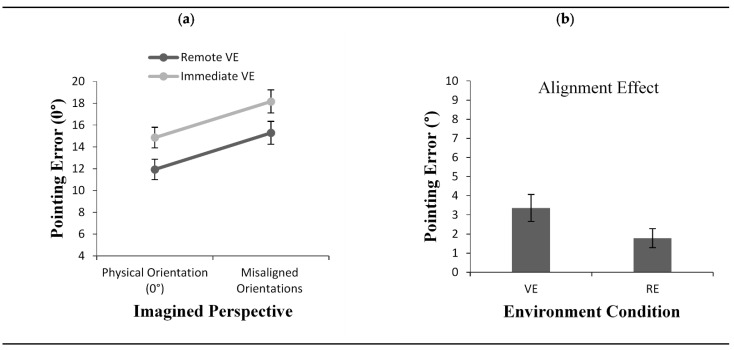
Pointing error (**a**) as a function of imagined perspective and alignment effect for pointing error (**b**) across test conditions (Remote VE vs. Immediate VE) in Experiment 1 and Experiment 2. Error bars represent standard errors from the ANOVA and from a *t*-test, respectively.

The results from the ANOVA revealed a main effect of imagined perspective, *F*(1, 46) = 45.38, *p* < 0.001, *η*^2^ = 0.49. As shown in Figure 5a, the error was smaller when the imagined perspective at testing was aligned with participants’ physical perspective (0°) compared to the remaining misaligned imagined perspectives. There was also a main effect for environment condition, with participants making larger errors in the Immediate VE condition (M = 16.50) than in the Remote VE condition (M = 13.61), *F*(1, 46) = 4.77, *p* < 0.05 *η*^2^ = 0.09. The alignment effect across the two conditions was equal (Figure 5b), as corroborated by the absence of an interaction between environment condition and imagined perspective, *F*(1, 46) = 0.002, *p* = 0.96, *η*^2^ = 0.00.

#### 3.2.2. Response Latency

**Figure 6:**
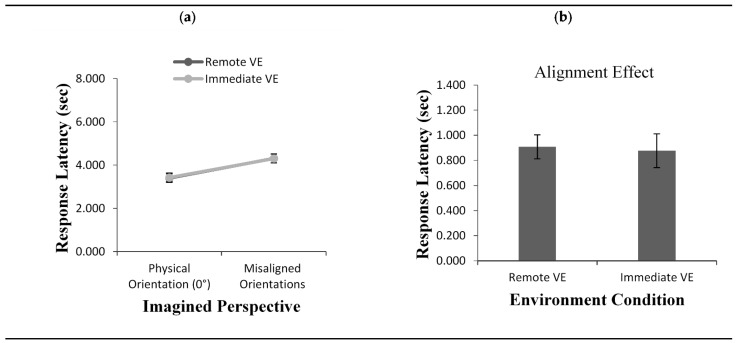
Response latency (**a**) as a function of imagined perspective and alignment effect for response latency (**b**) across test conditions (Remote VE vs. Immediate VE) in Experiment 1 and Experiment 2. Error bars represent standard errors from the ANOVA and from a *t*-test, respectively.

The ANOVA on response latency also showed a main effect of imagined perspective, *F*(1, 46) = 117.03, *p* < 0.001, *η*^2^ = 0.71. Overall, participants pointed faster when the imagined perspective adopted at testing was aligned than misaligned with their physical orientation (Figure 6a). In contrast to pointing error, for response time, there was neither a main effect for environment condition, *F*(1, 46) = 0.001, *p* = 0.97, *η*^2^ = 0.00, nor an interaction between environment condition and imagined perspective, *F*(1, 46) = 0.037, *p* = 0.84, *η*^2^ = 0.00, resulting in alignment effects of equal size across the two environment conditions (Figure 6b).

### 3.3. Discussion

As in Experiment 1, the results from Experiment 2 revealed an alignment effect: participant performance was better when they pointed from an imagined perspective that was aligned with their physical orientation than perspectives that were misaligned to it. Moreover, accuracy was lower in the Immediate VE condition of Experiment 2 than the Remote VE condition of Experiment 1. More importantly though, the size of the alignment effect did not differ between the two VE conditions.

Based on previous findings, we hypothesized that being immediate to a spatial scene, albeit external to it, might lead to a greater alignment effect than when reasoning about the same scene depicted on a screen. However, the present results revealed similar alignment effects across the two conditions we tested. This finding suggests that independent of whether the external observer is immediate or remote to the scene, in both scenarios, he/she encounters difficulties when pointing from imagined perspectives. This raises the question, what could account for this finding?

One possibility is that spatial conflicts were equally present in the two conditions if both conditions provided an immersive experience to participants. Although, in the Remote VE condition, we expected that participants would be more disengaged from the depicted scene and thus experience weaker sensorimotor interference, it is possible that this was not the case. The fact that, in both conditions, participants could not see their own body might have rendered the distinction between the scene and their body less salient.

Following this account, another possibility is that egocentric encoding takes place independent of whether the observer experiences an immediate layout or a remote one. The sensorimotor interference hypothesis states that conflicts when responding from imagined perspectives arise due to knowing the actual locations of objects. In order to compute and execute a response from a misaligned perspective, an observer needs to suppress these egocentric codes that define objects locations relative to the self. It might be the case that, in spatial perceptual tasks, these codes are hard to ignore, especially in immersive situations since stimuli are perceptually available and, therefore, the observer is always aware of their locations. This might not be true, however, in situations in which perceptual stimuli are not directly perceived as immersive, as in the case of Sohn and Carlson [6]. In their experiment, the alignment effect was eliminated with a non-spatial response mode, potentially by removing the sensorimotor conflicts associated with a spatial response.

Based on the latter observation, it could also be that strong sensorimotor conflicts were present in both conditions due to the nature of the response mode. In past studies, participants responded by deflecting a joystick (e.g., Avraamides et al. [13]), which may be less body-dependent than the response mode used in the present experiment. It is possible that, pointing to objects from an imagined perspective by extending one’s arm causes much sensorimotor interference during response execution because of the discrepancy in pointing direction between the actual position and the imagined position.

We designed Experiment 3 to examine this latter possibility. Specifically, in order to investigate whether the alignment effects documented were caused, at least partially, by the strong body-dependence of the response mode we used, in Experiment 3 we compared pointing towards objects locations by deflecting a joystick vs. by extending the arm. As it was technically challenging to create a VE condition in which participants would point with a joystick and have visual access to the response medium, we ran the joystick condition in the real world, aware of the fact that Experiment 1 showed a smaller alignment effect in the real world than in VE. In Experiment 3, we ran a *RE-Joystick* condition and compared performance to the RE condition of Experiment 1.

## 4. Experiment 3

As in the RE condition of Experiment 1 (for clarification purposes and for better discrimination across comparisons between Experiment 1 and Experiment 3, we will refer to the RE condition of Experiment 1 as “RE-Arm condition”), participants in Experiment 3 stood in the middle of the laboratory and viewed the layout on an actual projection screen. In this condition, though, participants responded by deflecting a joystick that was placed in front of them (RE-Joystick condition, Figure 6). If sensorimotor conflicts in the previous experiments were caused by the strong body-dependence of the response medium, the alignment effect should be smaller in the joystick than the arm pointing condition.

### 4.1. Method

#### 4.1.1. Participants

Twenty-six students from the University of Cyprus participated in the experiment in exchange for course credit. None of the participants had participated in Experiments 1 or 2.

#### 4.1.2. Materials and Procedure

**Figure 7:**
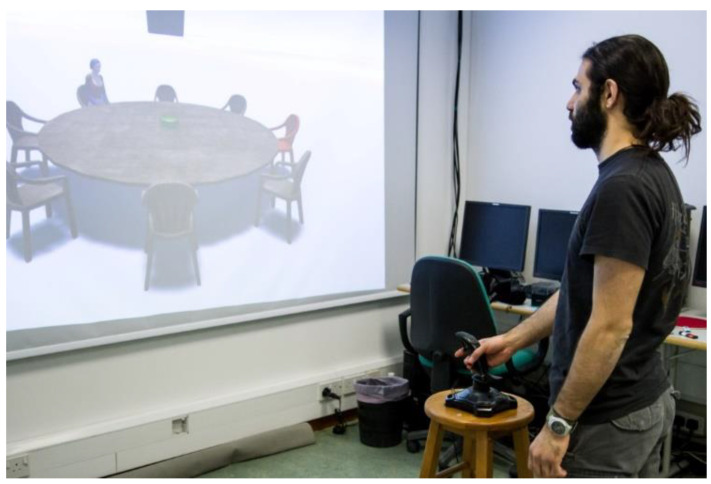
Example of a participant carrying out the task in the RE-Joystick condition of Experiment 3.

Materials were identical to the RE-Arm condition of Experiment 1 and the procedure followed the previous experiments, with one notable exception; while in Experiment 1 participants responded by extending their arm, in Experiment 3, they pointed by deflecting a joystick that was placed in from them on a stool (Figure 7). Joystick pointing responses reflected simple response mapping. For instance, when the target was at 45° to the left of the imagined perspective, participants had to move the joystick 45° to the left of the joystick’s forward orientation.

### 4.2. Results

In order to examine the effect of response mode on performance, we analyzed, together, the data from the RE-Joystick condition of Experiment 3 and the data from the RE-Arm condition of Experiment 1 (RE-Arm). Due to multiple testing using the same data set of Experiment 1, a Bonferroni correction was applied. As in previous experiments, data were analyzed separately for pointing error and response latency, with imagined perspective as the within participant factor and environment condition (Remote RE-Arm vs. Remote RE-Joystick) as the between participant factor, in Mixed Model ANOVAs. Pointing errors greater than 75° and response latencies deviating more than 3 standard deviations from the mean of each participant were removed from the analyses.

#### 4.2.1. Pointing Error

**Figure 8:**
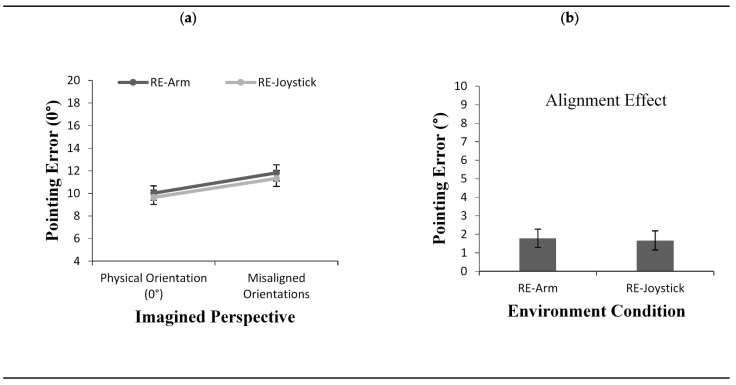
Pointing error (**a**) as a function of imagined perspective and alignment effect for pointing error (**b**) across test conditions (RE-Arm vs. RE-oystick) in Experiment 2 and Experiment 3. Error bars represent standard errors from the ANOVA and from a *t*-test, respectively.

The analysis comparing pointing error between the RE-Arm condition of Experiment 1 and the Remote RE-Joystick condition of Experiment 3 showed a main effect of imagined perspective, *F*(1, 50) = 23.21, *p* < 0.001, *η*^2^ = 0.31. The expected alignment effect was found with participants pointing more accurately from the perspective that was aligned than misaligned with their physical orientation in space (0°) (Figure 8a). However, results revealed neither a main effect for environment condition nor an interaction between environment condition and imagined perspective, *F*(1, 50) = 0.235, *p* = 0.63, *η*^2^ = 0.00 and *F*(1, 50) = 0.025, *p* = 0.87, *η*^2^ = 0.00, respectively. As seen in Figure 8b, the alignment effect was equal across the two testing conditions.

#### 4.2.2. Response Latency

**Figure 9:**
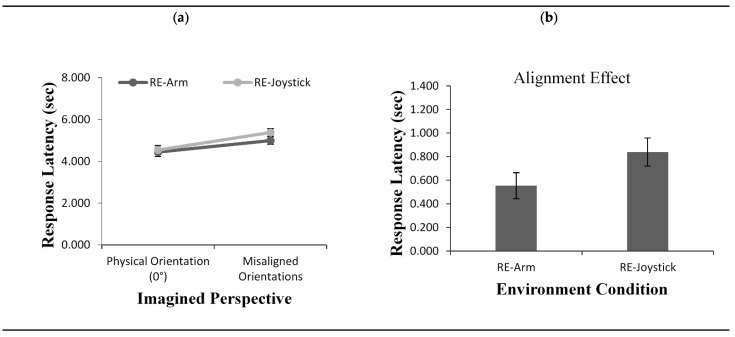
Response latency (**a**) as a function of imagined perspective and alignment effect for response latency (**b**) across test conditions (RE-Arm vs. RE-Joystick) in Experiment 2 and Experiment 3. Error bars represent standard errors from the ANOVA and from a *t*-test, respectively.

As with pointing error, response latency also showed a significant main effect of imagined perspective, *F*(1, 50) = 73.81, *p* < 0.001, *η*^2^ = 0.59. As shown in Figure 9a, participants were faster to respond from an aligned than a misaligned imagined perspective. Again, neither a main effect for condition nor an interaction between imagined perspective and environment condition were found (*F*(1, 50) = 0.857, *p* = 0.35, *η*^2^ = 0.01 and *F*(1, 50) = 3.082, *p* = 0.08, *η*^2^ = 0.05, respectively), although there was a trend towards a larger alignment effect when responding with a joystick (Figure 9b).

#### 4.2.3. Correlations with Paper-and-Pencil Measures

Correlational analyses were conducted to examine the relation between performance and perspective-taking skills as measured with the SOT and the SBSOD. The analysis included participants from all experiments to increase statistical power (N = 100). These analyses aimed to examine whether performance in the computerized perspective-taking task we used in the current experiments is related to individual differences in perspective taking as captured by the SOT [18]. As shown in Table 1, participants’ overall error correlated positively with accuracy on the SOT, *r* = 0.53, *p* < 0.001. This result suggests that the novel task used in the current experiments can indeed capture differences in perspective taking ability. However, no significant correlation was found between performance in the experiment and scores on the SBSOD.

### 4.3. Discussion

As with previous experiments, the results from Experiment 3 showed a clear alignment effect with that participants performing better from imagined perspectives aligned than misaligned to their physical orientation. Importantly, the size of the alignment effect and performance overall were similar for when participants pointed by outstretching their arm or by deflecting the joystick. This finding shows that even if pointing with the arm is more dependent on one’s body than pointing with a joystick, it does not create any additional difficulties for reasoning from imagined perspectives. Thus, it seems unlikely that the alignment effect in Experiments 1 and 2 was caused by the response mode. Our conjecture is that the difficulties are driven by the salient self-to-object-codes that are hard to be ignored in a perceptual task. We discuss this possibility further in the General Discussion.

## 5. General Discussion

Several studies in spatial cognition have systematically examined the difficulties that are associated with adopting imagined perspectives [1,2,3,4,6,8,10,13,25]. Findings from these studies converge in that performance in perspective taking is inferior for perspectives that are misaligned, compared to those that are aligned, to one’s actual orientation. The presence of clear alignment effects in all conditions in the three experiments reported here corroborates these past findings.

Although a strong alignment effect in both accuracy and latency was observed regardless of the details of the testing situation, our main aim was to examine whether blocking visual access to one’s body and surrounding context would reduce these difficulties by decreasing or eliminating sensorimotor interference. The results of Experiment 1 indicated that the opposite was the case: the alignment effect was larger when participants were tested in a virtual than a real environment. Furthermore, participants were overall faster, albeit less accurate, when they carried out the task in a virtual rather than in a real environment. In addition, Experiment 2 showed that being an external observer to an immediate spatial scene presented in VR does not create additional difficulties for perspective taking compared to viewing the scene projected on a two-dimensional projection in VR. Finally, Experiment 3 indicated that pointing with the arm does not introduce any further difficulties for pointing from imagined perspectives.

Numerous studies have used VR as a tool for examining large-scale space perception. However, unlike the real world where our perceptual system automatically receives countless sensory information, perception of a virtual environment is dependent on the combination of sensory cues employed in the environment [26]. One advantage of VR is the control and manipulation of environmental and body cues within the virtual world [27]. The quality and richness of these cues can affect the way people interact with the mediated environment and whether they behave as if it was real (e.g., distance estimation [28,29]). Based on this account, we hypothesized that limited access to these cues, as is frequently the case in virtual worlds compared to real worlds, might affect spatial reasoning from imagined perspectives. Although our hypothesis was that the alignment effect would be smaller in the virtual than in the real environment due to reduced saliency of self-to-object relations, our results revealed the opposite effect: the alignment effect was smaller when participants viewed the layout on a real projector screen compared to the virtual world. Although this could be a general effect of viewing real vs. virtual stimuli, this possibility seems unlikely given that previous studies document no substantial differences in spatial perspective taking in the virtual vs. the real world [30].

One could also argue that the difference in performance between the real and the virtual world in Experiment 1 could be a result of differences in the sizes of stimuli used across the two conditions [31]. In the real-world condition, the layout was bigger in size than in the virtual world, which could have potentially lead to misperceptions of depth in VR and in real world. These misperceptions could result in distorted estimates of the relative locations of targets and hereafter, in differences in pointing performance. For instance, pointing towards a target located at 45° on a small table would require different calculation of pointing angle compared to pointing to the same target on a large table. An earlier study, however, demonstrated that large displays allow for a wider field of view that increases the feeling of presence, immersion and also improves performance in certain spatial tasks such as navigation [32]. Therefore, better performance in the real world condition of our experiment could result from better exploration of the scene and not from differences in the sizes of stimuli.

A more interesting possibility is that the distinction between the self and the layout becomes even more prominent when visual information about the position of the observer’s body is available. That is, it could be that viewing one’s body at a certain location helps to anchor them to the real world and detach them from the spatial scene. In contrast, when visual access to the body is lacking—as is typically the case in immersive VR—the boundaries between the self and the environment are blurred and immersion is increased, which may cause greater sensorimotor conflicts than in real situations where information about the body is available.

This possibility is in line with past findings showing that visual awareness of one’s body helps establishing a reference frame about the person’s current position in space and increase performance for egocentric tasks (i.e., tasks requiring one to reason from their actual perspective). For example, research on distance judgments showed that participants made more accurate estimates in VR when they viewed representations of their bodies than when they did not [33,34]. In general, body representations are known to enhance the feeling of presence in VR [35,36,37] and, as such, they might be a source of stronger sensorimotor conflicts. Indeed, past research has shown that sensorimotor interference (as inferred by greater error and latency when responding from imagined perspectives) is reduced when one is decontextualized from the spatial scene [24] or is disoriented before testing [38], presumably because, in these cases, the spatial relation between one’s body and the spatial scene is irrelevant or unknown.

A striking result from the present study is that the size of the alignment effect in Experiment 2 was the same regardless of whether participants viewed the spatial scene as part of the immediate environment vs. as a projected remote scene. As mentioned above, previous studies have shown that alignment effects are less pronounced when reasoning about immediate than remote spatial layouts [13,24]. Following up on these studies, in Experiment 2 we examined whether this would also be the case when the observer is external to an immediate scene. Based on the past literature, we hypothesized that the immediate scene would induce greater sensorimotor conflicts, yielding a larger alignment effect than a scene presented on a 2D projection screen. The 2D projection screen has a small field of view that limits the perceptual affordances with the environment and makes a clear distinction between observer and scene. On the other hand, the immediate scene has a wider field of view, thus eliciting higher levels of immersion [21,39], and provides the feeling that the observer is part of the layout. Still, the results did not support our hypothesis: we found no difference in the size of the alignment effect when participants pointed towards object locations in the immediate and the remote virtual environments. What could account for this finding?

Our conjecture is that the explanation relates to the perceptual nature of the task used. Although previous studies in spatial cognition indicate that sensorimotor interference is less pronounced when reasoning about remote environments [10], their conclusions come from memory tasks in which egocentric relations might be easier to ignore. Perhaps, in perceptual tasks where objects are constantly available, self-to-object codes exert an influence even when the observer is not part of the spatial layout. Indeed, a long tradition of research in the field of Stimulus–Response Compatibility (SRC) documents the presence of interference from self-to-object codes for stimuli presented on a screen [14,40,41,42]. Perceptual tasks, like the one we used here, may produce spatial conflicts of the kind observed in SRC studies, where what matters is the spatial correspondence between stimulus and response locations. If this is the case, an interesting direction of future work would be to examine whether sensorimotor interference in perceptual tasks, such as the one used here, can be overcome following extensive practice with the task.

An alternative explanation that we cannot rule out is that participants faced no sensorimotor interference in either of the VR conditions. This could be the case if being external to a spatial scene, and perceiving all objects at once, relies more on allocentric than egocentric encoding. Although several researchers have argued that egocentric and allocentric encoding takes place simultaneously when experiencing a spatial layout ([43,44] see Avraamides and Kelly [11] for a review of theories with multiple systems of encoding), perspective taking from an external perspective may be carried out solely on the basis of allocentric information. In this case, the alignment effect observed in both VR conditions could be attributed to mental transformation processes, reflecting the time and error associated with adopting the imagined perspective [4].

Although our findings cannot differentiate between the accounts outlined above, future studies manipulating whether the observer is external or immersed in the scene may shed light on the source of the alignment effect. Here, we can simply note that, unlike what is expected from the results of previous studies using memory tasks with observers immersed in the spatial scene, our results show that the size of the alignment effect might not be affected by whether the scene is perceived by an external observer in a perceptual task as immediate or remote.

Overall, the findings from the present study add to the literature on perspective taking by examining conditions that, to our knowledge, have not been investigated before. In particular, they showed that the alignment effect is greater in the virtual than in the real world, a finding that may have important implications for future studies in spatial cognition, in which VR is an increasingly popular tool, but also for the design of games and other VR applications that require reasoning from different perspectives. Although our conjecture is that this finding is related to having no visual access to one’s body in the VR conditions we tested, future research may look into the source of this effect, perhaps by including conditions in which a virtual body or a virtual arm is available in VR. Alternatively, future experiments could examine whether lack of visual access to body information reduces difficulties in perspective taking by blocking vision of participants’ arms in the real world condition.

## Figures and Tables

**Table 1:** Correlations and Descriptive Statistics for variables of Experiment 1 and 2.

	M(SD)	SOT	Resp.Lat.	Align.Eff.	P.Error	SBSOD
SOT	43.69 (31.35)	1	0.05	−0.08	0.53 ***	−0.04
Resp.Lat.	4.66 (1.04)		1	0.03	−0.08	0.04
Align.Eff.	0.79 (0.58)			1	0.00	0.08
P.Error	16.12 (6.84)				1	−0.09
SBSOD	4.19 (0.96)					1

Ns = not significant (*p* > 0.05), *** *p* < 0.001. Align.Eff. = Alignment Effect, SOT = Spatial Orientation Test, P.Error = Pointing Error, Resp.Lat. = Response Latency, SBSOD = Santa Barbara Sense of Direction Scale.

## Data Availability

All data are available by contacting the author.
